# A Validated Set of Ascorbate Peroxidase-Based Organelle Markers for Electron Microscopy of Saccharomyces cerevisiae

**DOI:** 10.1128/msphere.00107-22

**Published:** 2022-06-21

**Authors:** Hui Li, Cheng-Wen He, Jing Zhu, Zhiping Xie

**Affiliations:** a State Key Laboratory of Microbial Metabolism and Joint International Research Laboratory of Metabolic and Developmental Sciences, School of Life Sciences and Biotechnology, Shanghai Jiao Tong University, Shanghai, China People’s Republic of China; University of Georgia

**Keywords:** organelle, ascorbate peroxidase, fluorescent protein, electron microscopy, immunofluorescence, yeast

## Abstract

Genetically encoded tags, such as engineered ascorbate peroxidase APEX2, offer unique advantages for the specific labeling of subcellular structures in electron microscopy (EM). However, the use of APEX2 in EM investigation of yeast has been limited. Here we describe the development of APEX2-based organelle markers for Saccharomyces cerevisiae. We found that with regard to APEX2 -catalyzed formation of diaminobenzidine precipitation, cell wall removal was not essential during sample preparation, yet the presence of fluorescent proteins in APEX2 chimeras had a negative impact. We showed that major organelles including endoplasmic reticulum, early Golgi, late Golgi/early endosomes, late endosomes, mitochondria, peroxisomes, and lipid droplets could be labeled by appropriate APEX2 chimeras. The subcellular localization of our APEX2 chimeras was verified by EM visualization and supplemented with immunofluorescence colocalization analysis when necessary, validating their feasibility as organelle markers.

**IMPORTANCE** Yeast is an excellent single cellular model system for studying basic cellular processes. However, yeast cells are much smaller than most animal and plant cells, making the observation and recognition of yeast subcellular structures challenging. Here we developed a set of yeast organelle markers for use in electron microscopy and documented our technical approach for using this method.

## INTRODUCTION

Electron microscopy (EM) is a powerful tool in extracting structural information below the resolution limit of light microscopy. However, it is generally less versatile than light microscopy when it comes to specific labeling of a certain structure or a certain molecule. The use of fluorescent proteins in live cells and immunofluorescent labeling in fixed cells is routine in light microscopy. In contrast, the use of most fluorescent proteins in EM is hampered by the severe reduction of fluorescence by common chemical fixation and embedding procedures (1). One way to alleviate this limitation is the use of frozen samples in correlative light and electron microscopy (CLEM). However, the high hardware cost means it is out of reach for many researchers. Immunolabeling in EM, mostly in the form of immunogold labeling, has been successfully utilized for years. In this type of approach, compromises are often made to trade the preservation of ultrastructural detail for the preservation of antigenicity (2, 3). The fixative for immuno-EM is generally limited to mild aldehyde recipes. Osmium tetroxide and potassium permanganate, which can otherwise serve as fixatives and contrasting agents in conventional EM preparations, are avoided. As a result, for immuno-EM samples prepared with chemical fixation, the fidelity of organelle morphology and the visibility of membrane structures leave much to be desired.

Over the years, multiple genetically encoded EM tags have been developed. Recent examples include miniSOG (a plant flavoprotein derivative) (4), FerriTag (based on metal-containing ferritin particle) (5), engineered metallothioneins (6), and several plant peroxidase derivatives (7–10). Like fluorescent proteins, these tags are expressed by the host cell, usually as a part of a protein chimera. Since they do not rely on antibody-antigen interaction, these new tools are free from some of the technical constraints in conventional immuno-EM. Among the genetically encoded EM tags, engineered ascorbate peroxidases have the advantage that relatively few modifications over conventional EM preparation procedure are required for its application. The tag specific stain is initially generated as precipitation of diaminobenzidine (DAB) polymers, in a chemical reaction performed after aldehyde fixation. DAB polymers then attract heavy metals during subsequent processing and become visible in EM.

APEX2, an improved variant of engineered ascorbate peroxidase, has been successfully used in many model systems (11). Reports of its use in yeast EM studies, however, have been limited so far (12–14). Here we describe the development of a set of APEX2-based organelle markers for yeast, and document our technical approach for working with this tag.

## RESULTS

### Towards successful application of APEX2-based tags in EM.

To create a set of EM suitable markers, we built a collection of potential organelle markers from our validated fluorescent protein constructs (15), screened for those capable of producing good DAB staining by light microscopy, and experimented with EM sample preparation procedures. Instead of going into all the technical permutations, here we summarize several categories of observations we made during our experimentation with this method that may be of some value to researchers aiming to further optimize APEX2 sample preparation or to label other proteins of interest with APEX2.

**FIG 1:**
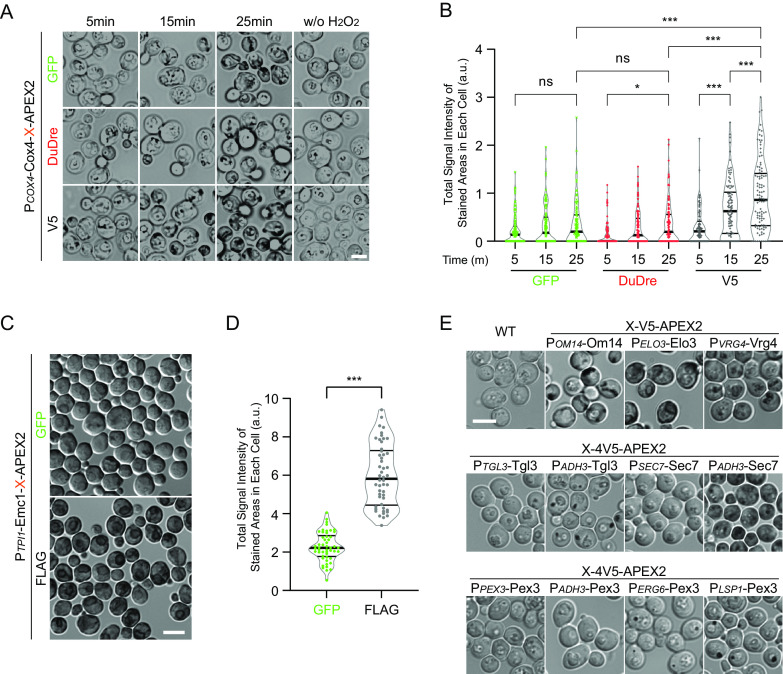
On diaminobenzidine (DAB) staining: fluorescent proteins and construct expression levels. (A and B) Presence of fluorescent proteins in Cox4-APEX2 chimeras hampers DAG staining. (A) Representative bright field (BF) images of cells after the indicated duration of staining and of a control group with hydrogen peroxide omitted. DuDre is a red fluorescent protein variant containing two DsRed-Express2 in tandem; w/o, without. (B) Total signal intensity in stained areas of each cell, displayed as truncated violin plots, with medians and quartiles marked. For each sample, 100 cells were manually marked for quantification; GFP, green fluorescent protein; a.u., arbitrary units. Select Tukey’s test pairwise results are displayed on top: ***, *P* < 0.01; *, *P* < 0.05; ns, not significant. (C and D) Presence of a fluorescent protein in Emc1-APEX2 chimera hampers DAG staining. (C) Representative differential interference contrast (DIC) images of cells after staining. (D) Total signal intensity in stained areas of each cell, displayed as truncated violin plots, with medians and quartiles marked. For each sample, 50 cells were manually marked for quantification. ***, *P* < 0.01, *t* test. (E) Good DAB staining generally requires protein expression level of 1 × 10^4^/cell or higher. Shown are representative DIC images of wild-type (WT) cells or cells carrying the indicated chimeras after staining. For some proteins, expression by their own promoter was sufficient to produce good DAB staining (OM14, Elo3, and Vrg4). For others, switching to *ADH3* promoter or stronger was necessary. Scale bar, 5 um.

The first category concerns the negative impact of fluorescent proteins. It is an attractive idea to tag a protein with both a fluorescent protein and APEX2, which theoretically allows dual use in both fluorescence microscopy and EM. It was possible to get it working (13, 14). However, the generation of DAB staining tended to be suboptimal in our experience. Furthermore, we noticed that the DAB staining procedure strongly reduced fluorescence signal. These observations prompted us to test if there is some type of interplay between a fluorescent protein and an ascorbate peroxidase. We examined five constructs based on two proteins, Cox4 and Emc1, labeling mitochondria inner membrane and endoplasmic reticulum, respectively. In both cases, replacement of small epitope tags (V5 or FLAG) with fluorescent proteins resulted in substantial declines of DAB precipitation level (Fig. 1A to D). Both green and red variants of fluorescent proteins were inhibitory. We therefore choose to eliminate fluorescent protein modules in APEX2 chimeras to improve staining.

The second category concerns the abundance of target proteins. We screened our candidate constructs for those capable of producing good DAB staining by light microscopy. Based on the published protein abundance data (16), it appeared that for most proteins we tested, good staining was achieved with expression levels of 1 × 10^4^ molecules per cell or higher (Fig. 1E). For proteins with endogenous expression substantially lower than that, switching to *ADH3* promoter (1.4 × 10^4^ molecules per cell) was generally sufficient. Pex3, a peroxisomal membrane protein, was one exception. It required going to *LSP1* or *ERG6* promoters (> 2 × 10^4^ molecules per cell) for good staining.

**FIG 2:**
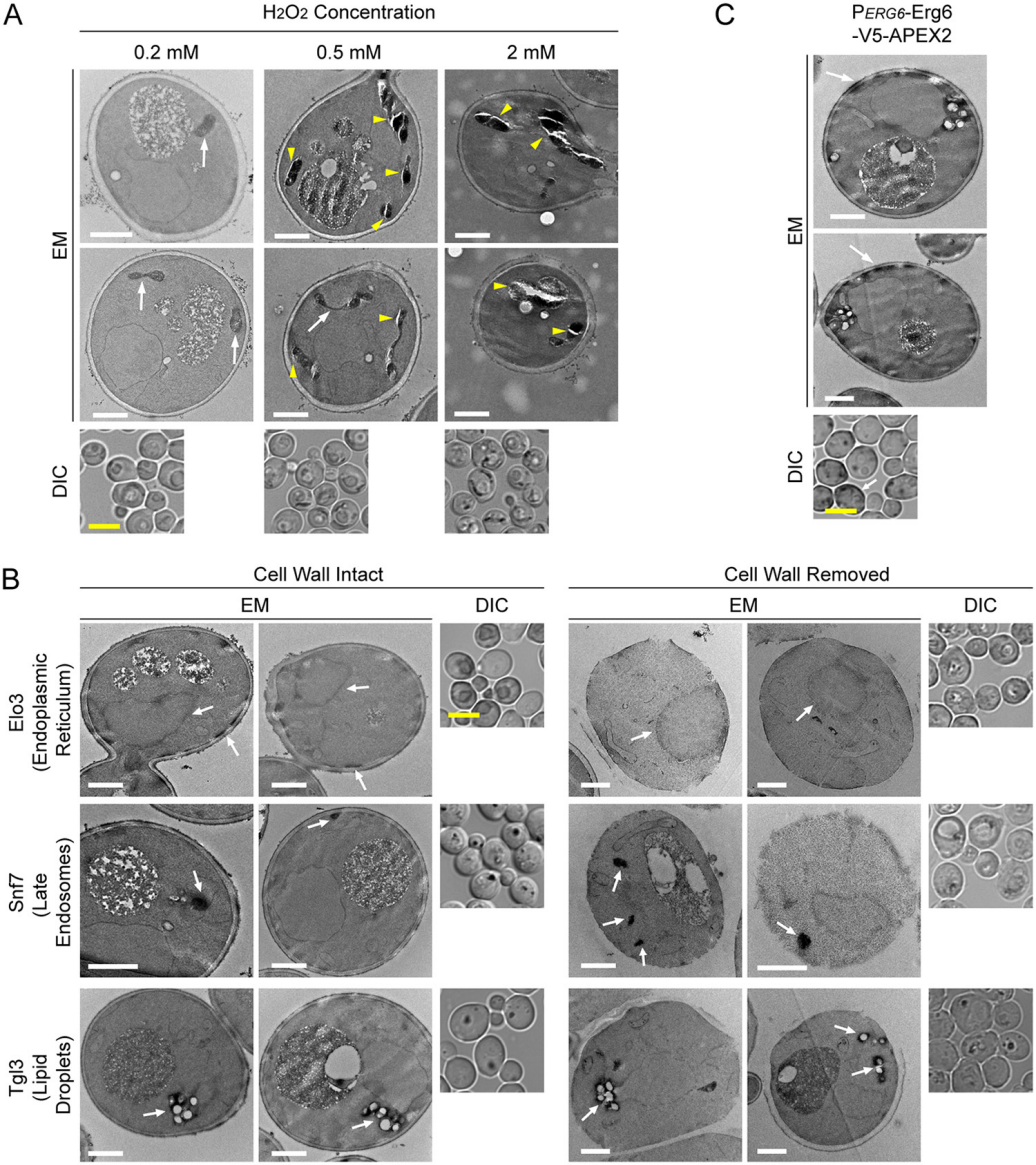
On resulting electron microscopy (EM) samples: hydrogen peroxide concentration, cell wall removal, and a rare case of spill over staining. (A) High concentration of hydrogen peroxide during DAB staining compromises sample structure and obscures fine detail. Yeast cells expressing P*COX4*-Cox4-V5-APEX2 (labeling mitochondria) were chemically fixed, stained by DAB, and then processed for transmission EM. Correlation between staining intensity and hydrogen peroxide concentration was visible in both light microscopy and EM. Fractures along stained membrane (yellow arrowheads) were frequent in samples treated with higher concentration of hydrogen peroxide. White arrows, mitochondria with acceptable structural preservation. (B) Cell wall removal is not necessary for the use of APEX2 in EM. Yeast cells were processed for EM in the same procedure, except that in one group enzymatic digestion of cells was performed (after chemical fixation, before potassium permanganate treatment). The inclusion of this step did not bring noticeable improvement. Arrows, organelles stained by DAB. (C) In rare cases, DAB staining may occur in unintended locations. Erg6-V5-APEX2 was the only exception among all the chimeras we tested. Erg6 is supposedly a lipid droplet protein. In EM, staining at the cell periphery was also present. Arrows, DAB staining in cell periphery. White scale bar in EM images, 1 um. Yellow scale bar in DIC images, 5 um.

The third category concerns EM sample preparation optimizations, i.e., variations in the protocol that made a difference in the final outcome. Hydrogen peroxide is an essential reactant in APEX2 -catalyzed production of DAB polymers. High concentrations of hydrogen peroxide (2 mM or above) promoted strong DAB staining. However, this condition brought two undesirable effects (Fig. 2A). One is the blurring of ultrastructural details. The other is the weakening of resin integrity, with fractures frequently occurring along the stained membrane and whole pieces of organelles disappearing in ultra-thin sections. We found that for most organelle markers, a hydrogen peroxide concentration of 0.2 mM provided a good balance between stain generation and structural preservation. In theory, many embedding resins are compatible with the APEX2 labeling. Among the three resins we tested, even though all worked fine for regular EM, only LR White yielded good embedding for DAB stained yeast samples. Samples prepared with Epon 812 or Spurr’s resin tended to have large pieces of cytoplasm missing after sectioning (data not shown), presumably resulting from suboptimal penetration of DAB-stained cells. Lastly, we noticed that the freshness of the fixative was essential for efficient DAB staining. Performance degradation became obvious after 1 week of storage at 4°C.

The last category concerns EM sample preparation simplifications. Some existing reports of using APEX2 in yeast for protein proximity labeling or EM visualization adopt a step of cell wall enzymatic digestion in their sample preparations (12, 17). In our experience, the removal of cell wall brought no discernible benefits to the final outcome of EM images (Fig. 2B). Another dispensable step we found is the quenching of unreacted aldehyde with glycine after fixation. We therefore arrived at a simplified procedure with these two steps omitted. We also found that as the buffer base for fixation, washing, and DAB staining solutions, phosphate-buffered saline (PBS) was sufficient, with alternatives including cacodylate buffer, Dulbecco’s PBS, and PHEM (a buffer containing PIPES, HEPES, EGTA, and MgCl_2_) yielding comparable results.

### APEX2-based organelle markers: typical outcome in EM, verification of subcellular localization, and assessment of functional impacts.

**FIG 3:**
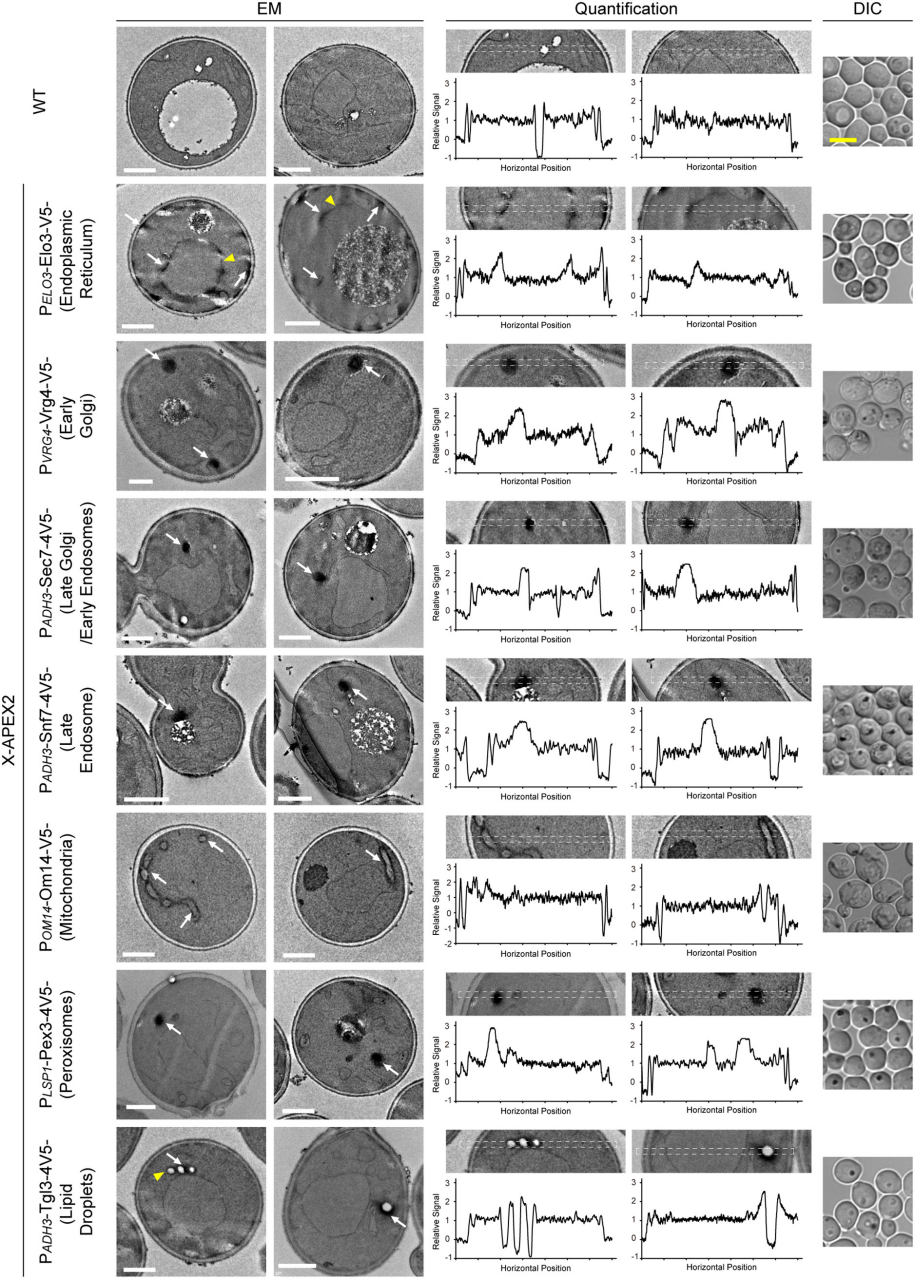
Representative EM images of yeast cells. Midlog phase wild-type yeast cells or those expressing the indicated APEX2 chimeras were chemically fixed, stained by DAB, and then processed for transmission electron microscopy. For each strain, two representative EM images, two strips of images that were quantified, line graphs of normalized intensity along the quantified image strips, and a DIC image of cells prior to EM fixation are shown. The quantified strips are marked by dashed boxes. For normalization, the average intensity of the extracellular space was set to 0, and the average intensity of the cytosol to 1. In control wild-type cells, the relative intensities of recognizable structures were mostly in the 1.4 ~1.9 range. In APEX2 labeled samples, the relative intensity of labeled structures were mostly in the 2.0 ~2.8 range. (Please see main text for more detailed breakdown). White scale bar in EM images, 1 um. White arrows, organelles strongly stained by DAB. Yellow arrowheads, potentially the same type of organelle mildly stained by DAB. Yellow scale bar in DIC images, 5 um.

After screening by light microscopy, we then tested the performance of candidate markers in EM (Fig. 3). Preparation of APEX2 marked EM samples differs from conventional EM mainly in the addition of a staining routine before permanganate treatment. Therefore, many membrane structures are expected to be visible in control cells that do not express any APEX2 markers (wild type [WT] in Fig. 3). With the average intensities of extracellular space and cytosol normalized to 0 and 1, respectively, we found that for structures that we were comfortable in assigning an identity based on experience, including the endoplasmic reticulum, mitochondria, and plasma membrane, the intensities of the membrane profiles are mostly in the 1.5 ~1.7 range. The borders of lipid droplets were stained stronger, with intensities in the 1.8 ~1.9 range. The outer layer of the cell wall was also strongly stained.

The expression of an APEX2 chimera boosted the staining intensities of its target structure (white arrows in Fig. 3). For cells expressing Elo3-APEX2, the intensities of nuclear endoplasmic reticulum were mostly in the 1.9 ~2.3 range, and that of peripheral endoplasmic reticulum in the 2.5 ~2.7 range. For cells expressing OM14-APEX2, targeting the mitochondrial outer membrane, the intensities were in the 2.2 ~2.4 range. Both the endoplasmic reticulum and the mitochondria are large organelles with characteristic morphologies. Based on the morphological characters of stained structures, we consider Elo3-APEX2 and OM14-APEX2 to be suitable markers of the respective organelles. Among the small organelles, the light interior of lipid droplets allows them to be discerned from the cytosol. The staining of Tgl3-APEX2 were specifically confined to the surrounding of such structures, indicating that Tgl3-APEX2 properly labeled the lipid droplets. In this round of testing, we noted a particular case regarding Erg6. This is a lipid droplet protein, and our immunofluorescence confirmed the expected localization of its chimera (data not shown). However, in EM, Erg6-APEX2 stained not only lipid droplets, but also patches of cell periphery beneath the plasma membrane (Fig. 2C). Such “spill-over” was only observed for Erg6-APEX2, but not Tgl3-APEX2, another lipid droplet marker, or other organelle markers we tested. For stained membrane profiles, the intensity of the staining may be uneven along the membrane or among different entities of the same type of organelle. When the normalized staining intensity fell below 2 (yellow arrowheads in Fig. 3), some level of subjective evaluation was needed to categorize the structures.

**FIG 4:**
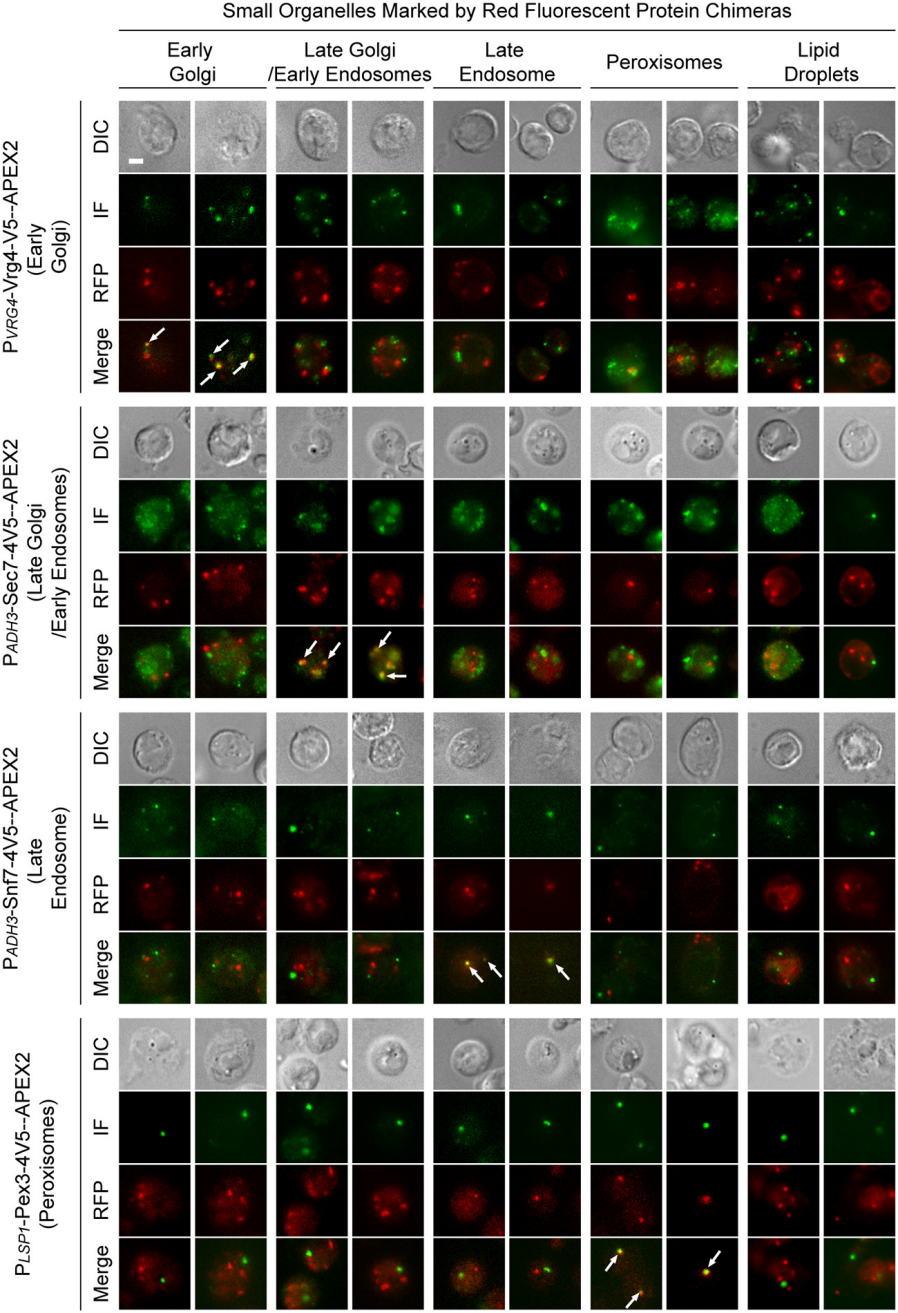
Verification of the subcellular localization of APEX2-based organelle markers by immunofluorescent microscopy. Midlog phase yeast cells expressing the indicated APEX2 construct, in combination with a red fluorescent protein (RFP)-based organelle marker, were processed for immunofluorescent microscopy. The RFP markers label the following organelles: Anp1-mCherry for early Golgi, Sec7-DuDre for late Golgi/early endosomes, Vps4-DuDre (in combination with all except for Snf7-APEX2) and Snf7-mCherry (in combination with Snf7-APEX2) for late endosomes, and Erg6-mCherry for lipid droplets. Representative single slices are shown. Green channel corresponds to immunofluorescence (IF) of the V5 tag in APEX2 chimeras. Arrows, colocalizated green and red signal. Scale bar, 2 um.

For the remaining small organelles, including the early Golgi, late Golgi/early endosomes, late endosomes, and peroxisomes, it is generally difficult to recognize them with strong confidence in conventional EM samples. With APEX2 labeling, we were able to locate clearly stained structures in the cytoplasm (Fig. 3). Under the aforementioned quantification scheme, the intensities of the profiles were in the following ranges: 2.4 ~2.8 for Vrg4-APEX2 (early Golgi), 2.2 ~2.5 for Sec7-APEX2 (late Golgi/early endosomes), 2.4 ~2.6 for Snf7-APEX2 (late endosomes), and 2.3 ~2.8 for Pex3-APEX2 (peroxisomes). We verified the fidelity of these four APEX2 markers by immunofluorescence in comparison with red fluorescent protein-based organelle markers (Fig. 4). Immuno-fluorescent puncta of Vrg4, Sec7, Snf7, and Pex3 constucts colocalized with the red signal of their intended organelles, but not those of other puncta organelles, indicating that the APEX2 chimeras were correctly targeted.

**FIG 5:**
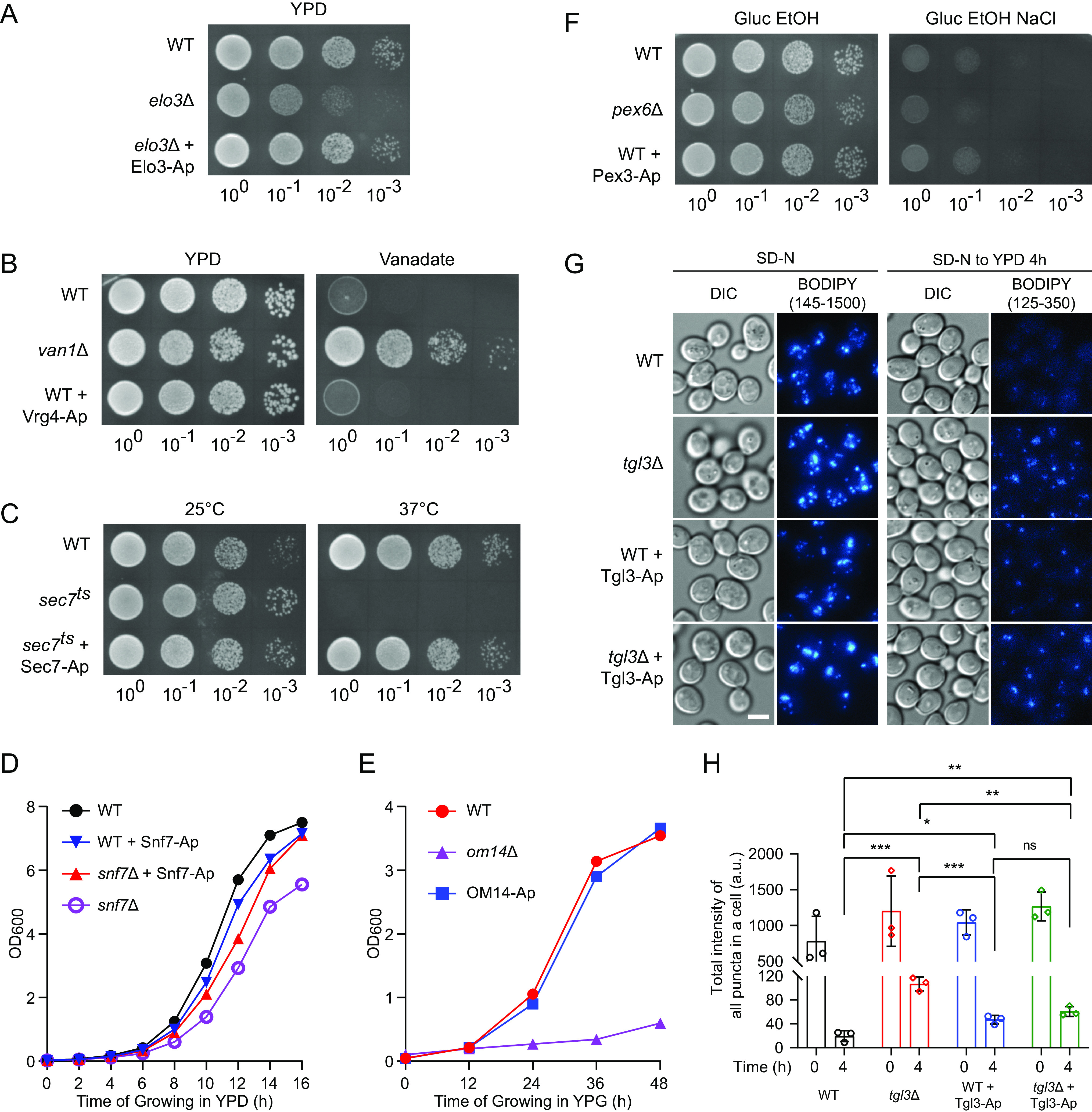
Functional impacts of expressing the APEX2-based organelle markers. Three types of assays were used to evaluate the functional impacts of expressing the indicated APEX2 (Ap) chimeras, either in the presence or the absence of the corresponding untagged genes in the genome: growth assays using solid agar plates, growth assays using liquid media, and an imaging-based lipolysis assay. For agar plate growth assays, the same optical densities (ODs) of midlog phase yeast cells were collected, from which serial dilutions were made and spotted on plates to grow for appropriate amounts of time. For liquid media growth assays, midlog phase yeast cells were diluted to the same starting OD and allowed to grow. OD values were monitored at the indicated time points. To observe lipolysis, yeast cells were first nitrogen-starved for 1 h to induce triacylglycerol production, then shifted to nutrient rich medium for 4 h to allow triacylglycerol consumption. The levels of cellular triacylglycerol was assessed by BODIPY staining and quantified. The results are presented as the following: evaluation of Elo3 function by agar plate growth assay (A); evaluation of Vrg4 function by agar plate growth assay (a functional Vrg4 confers sensitivity to vanadate) (B); evaluation of Sec7 function by agar plate growth assay (37°C is the nonpermissive temperature for the *sec7-ts* mutant) (C); evaluation of Snf7 function by liquid culture growth assay (D); evaluation of OM14 function by liquid culture growth assay (mitochondrial metabolic activity is critical for utilizing glycerol as the carbon source) (E); and evaluation of Pex3 function by agar plate growth assay (peroxisomal metabolic activity is critical for growing in ethanol medium containing high salt) (F). (A to F) Representative results from at least two independent repeats are shown. (G and H) Evaluation of Tgl3 function by lipolysis assay: representative microscopy images (G; scale bar, 5 um) and quantification from three independent repeats (H). YPD, Yeast extract / Peptone / Destrose time, hours passed since shifting back to rich medium; error bar, SD. Select Tukey’s test pairwise results displayed on top: ***, *P* < 0.01; *, *P* < 0.05; ns, not significant.

Lastly, we examined the functional impact of expressing APEX2 chimeras. Among the seven fusion partner genes, for five of them we were able to test if an APEX2 chimeras was functional as a replacement of the untagged version. Because of recently imposed logistical constraints, for Vrg4 and Pex3 fusions we could only assess whether the APEX2 constructs had dominant effects when expressed in the WT background. For six out of the seven organelle markers, their functionalities were evaluated by growth in normal nutrient rich condition or under selective pressure (Fig. 5A to F). Defects in *ELO3*, *SEC7*, and *SNF7* reduce or inhibit growth in nutrient rich conditions. Test for *SEC7* employed a *sec7-ts* temperature sensitive mutant, for which inactivation occurs at 37°C. The function of VRG4 was evaluated by testing sensitivity to vanadate (18). The functions of OM14 and PEX3 were evaluated in selective media testing mitochondrial and peroxisomal metabolic activities (19). The function of Tgl3, a triacylglycerol lipase, was evaluated by an imaging-based lipolysis assay (Fig. 5G and H). In conclusion, we found that constructs based on Elo3, Sec7, and OM14 were fully functional. Vrg4-APEX2 and Pex3-APEX2 did not introduce functional compromises when expressed in WT cells. Snf7-APEX2 was both partially functional and partially dominant negative. Tgl3-APEX2 was functional but had a moderate dominant negative effect when expressed in WT cells. Thus, when using Snf7-APEX2 and Tlg3-APEX2 to label late endosomes and lipid droplets, respectively, one will need to consider whether such levels of functional disturbance is tolerable in the actual research context. The properties of APEX2-based organelle markers are summarized in Table 1.

**TABLE 1:** List of APEX2-based organelle markers

Organelles	Verified markers and notable properties	Less ideal candidates
Endoplasmic reticulum	Elo3-V5-APEX2	P_TPI1_-Emc1-flag-APEX2: need 2 mM H_2_O_2_ to obtain discernable staining in EM^*a*^.
Early Golgi	Vrg4-V5-APEX2	
Late Golgi/early endosomes	P_ADH3_-Sec7-4V5-APEX2	
Late endosomes	P_ADH3_-Snf7-4V5-APEX2: partially functional and partially dominant negative; need 0.5 mM H_2_O_2_ to obtain good staining in EM.	
Mitochondria	OM14-V5-APEX2	Cox4-V5-APEX2: tendency to generate fracture along mitochondrial membrane in EM sections.
Peroxisomes	P_LSP1_-Pex3-4V5-APEX2	
Lipid droplets	P_ADH3_-Tgl3-4V5-APEX2: functional and moderately dominant negative.	Erg6-V5-APEX2: localization correct in immunofluorescence but additionally stains cell periphery in EM.

a EM, electron miscropy.

## DISCUSSION

In the present study, we developed a set of ascorbate peroxidase-based yeast organelle markers for use in EM, covering endoplasmic reticulum, early Golgi, late Golgi/early endosomes, late endosomes, mitochondria, peroxisomes, and lipid droplets. We also described several observations regarding chimera construct design and EM sample preparation that may aid future use and optimization of ascorbate peroxidase tags in yeast. Our tools can potentially be utilized in at least three scenarios. One is to gain familiarity with the morphology of particular organelles, after which investigations may be carried out without the labels. A second scenario pertains to several organelles with morphologies difficult to distinguish from each other. Golgi apparatuses, endosomes, and peroxisomes are all small single membrane structures. Without a specific label, their recognition is challenging even for experienced scientists. Lastly, a more intriguing scenario is where the change of organelle morphology is the subject of investigation, either caused by genetic or environmental alterations. In this scenario, without the aid of a proper label, excessive reliance on prior familiarity runs the risk of circular reasoning.

When used in the simplest form, the DAB precipitation generated by peroxidases manifests as diffuse staining around the structure the enzymes reside on, which paradoxically obscures the fine details sought after by many EM investigations. In principle, it is possible to optimize the staining parameters and manually pick cells with a light staining for subsequent analysis. However, below a certain threshold, it is difficult to quantitatively differentiate labeled structures from background membrane staining by heavy metals (Fig. 3). To address this limitation, further optimization of EM sample preparation have been developed. Mihelc et al. (20) demonstrated that in place of conventional chemical fixation, the use of high pressure freezing and freeze substitution allowed better structural preservation and detail resolution in APEX2 labeled membrane structures. More recently, Rae et al. (21) described a method that coverts the DAB polymer into gold particles, producing an immunogold-like effect and eliminating the obscuring issue all together for ascorbate peroxidas. Although these optimizations have not been tested in yeast, they are a testament to both the great potential and the continued development of EM methodology using peroxidase tags.

Our data demonstrate that APEX2 chimeras are generally applicable to most organelles in yeast. With further optimization, genetic EM tags may 1 day become as common in EM studies as fluorescent proteins are in live cell imaging and bring new opportunities of discovery for researchers using yeast and other model systems.

## MATERIALS AND METHODS

### Plasmids and strains.

Plasmids newly constructed for this study are listed in Table S1 to S3. Plasmids in Table S1 and S2 were constructed by the following steps: insertion fragment was obtained by PCR amplification, plasmid backbone was prepared by either restriction enzyme digestion (Table S1) or PCR amplification (Table S2), and the two pieces were combined by enzyme mediated recombination. Plasmids in Table S3 were constructed by enzymatic digestion of parental plasmids and ligase mediated ligation.

10.1128/msphere.00107-22.1 TABLE S1 Plasmid set I. For plasmids in this set, the insert fragments were obtained by PCR amplification, the plasmid backbones were obtained by enzymatic digestion, and the two parts were joined by enzyme-mediated recombination. Download Table S1, PDF file, 0.06 MB. Copyright © 2022 Li et al. 2022 Li et al. https://creativecommons.org/licenses/by/4.0/ This content is distributed under the terms of the Creative Commons Attribution 4.0 International license.

10.1128/msphere.00107-22.2 TABLE S2 Plasmid set II. For plasmids in this set, the insert fragments and the plasmid backbones were obtained by PCR amplification, and the two parts were joined by enzyme-mediated recombination. Download Table S2, PDF file, 0.06 MB. Copyright © 2022 Li et al. 2022 Li et al. https://creativecommons.org/licenses/by/4.0/ This content is distributed under the terms of the Creative Commons Attribution 4.0 International license.

10.1128/msphere.00107-22.3 TABLE S3 Plasmid set III. For plasmids in this set, the insert fragments and the plasmid backbones were obtained by enzymatic digestion, and the two parts were joined by enzyme-mediated ligation. Download Table S3, PDF file, 0.2 MB. Copyright © 2022 Li et al. 2022 Li et al. https://creativecommons.org/licenses/by/4.0/ This content is distributed under the terms of the Creative Commons Attribution 4.0 International license.

Strains used in this study are listed in Table S4. Integration plasmids were linearized by enzymatic digestion and transformed into yeast cells by the lithium acetate method (22).

10.1128/msphere.00107-22.4 TABLE S4 Strains used in this study. The genotypes of strains used in this study are listed. Download Table S4, PDF file, 0.3 MB. Copyright © 2022 Li et al. 2022 Li et al. https://creativecommons.org/licenses/by/4.0/ This content is distributed under the terms of the Creative Commons Attribution 4.0 International license.

### Yeast media.

Yeast media were as follows: YPD: 1% yeast extract, 2% peptone, and 2% glucose; YPG: 1% yeast extract, 2% peptone, and 3% glycerol; YPEG: 1% yeast extract, 2% peptone, 0.5% glucose, and 3% ethanol; YPEG + NaCl: YPEG with 1 M sodium chloride; SD-N: 0.17%YNB (yeast nitrogen base without amino acids and ammonium sulfate) and 2% glucose; and SMD: 2% glucose, 0.67% YNB (yeast nitrogen base without amino acids), 30 mg/L adenine, 30 mg/L lysine, 30 mg/L methionine, 20 mg/L histidine, 20 mg/L uracil, 50 mg/L tryptophan, and 50 mg/L leucine. Corresponding plates contained 2% agar.

### Sample preparation for transmission electron microcopy.

Yeast cells were collected by centrifugation (4,000 × *g*) and washed once by phosphate-buffered saline (PBS, pH 7.2). Cells were then suspended in fixation buffer (2% paraformaldehyde [Sangon-Biotech; A500684], 2.5% glutaraldehyde [Sangon-Biotech; A600875], prepared in PBS) and incubated at 30°C with 200 rpm shaking for 30 min. Subsequent centrifugation steps were performed at 2,000 g. The fixative was removed by washing with PBS for three times. DAB staining was performed by incubating fixed cells in staining buffer (1.4 mM 3,3′-diaminobenzidine [Sangon-Biotech; A600140], 10 mM H_2_O_2_ [Aladdin; H112517], 0.1% Triton X-100 [Sangon-Biotech; A110694], prepared in PBS) at room temperature, shielded from light, with 100 rpm rotation for 15 min. Staining was terminated by washing with PBS twice. At this stage, an aliquot of cells was checked by light microscopy to verify staining progress. Cells were then collected by centrifugation and incubated with 2% potassium permanganate (General-Reagent; P1363894) at room temperature for 2 h. Charred cells were washed five times in distilled water to remove potassium permanganate. (Note that cells turn black after permanganate treatment, and remain so after washing.) Dehydration was performed by sequential incubation with increasing concentrations of ethanol (once each at 30%, 50%, 70%, 80%, 90%, 95%, and twice at 100%) at room temperature for 15 min at each step. Resin infiltration was performed by sequential incubation at room temperature with LR White (TED PELLA; 18181)/ethanol (1:2 vol:vol) for 2 h, LR White/ethanol (2:1 vol:vol) for 12 h, and then twice with fresh 100% LR White for 12 h each. Resin was polymerized by incubation at 60°C for 48 h. 70 nm thin sections were prepared on a Leica UC7 ultramicrotome. Sections were picked up and laid on Formvar coated 200-mech copper grid (Zhongjingkeyi; AZH200). Sections were then stained with uranyl acetate alternative (TED PELLA; 19485) for 10 min at room temperature, rinsed with distilled water, and dried in air. This was followed by staining with 2% lead citrate (TED PELLA; 19312) for 6 min at room temperature in metal box containing sodium hydroxide tablets, providing a dark and carbon dioxide free environment, then rinsed and let dry.

### Quantification of transmission electron microcopy images.

A square region containing a target cell was manually selected and resized to 800 × 800 pixels. A strip of 700 × 15 pixels was manually picked, and the intensity profile (*x*) was measured using the “Plot Profile” tool in ImageJ. Extracellular signal (*a*) and cytosolic signal (*b*) were measured by randomly picking five 30 × 30 squares for measuring and averaging. The normalized intensity was computed as (*x* − *a*)/(*b* − *a*).

### Sample preparation for immunofluorescent microcopy.

Yeast cells were fixed as described in the previous section, then washed with sorbitol buffer (1.2 M sorbitol [Sangon-Biotech; A610491] in PBS). Cell wall removal was performed by incubation cells in spheroplasting buffer (1.2 M sorbitol, 1% β-mercaptoethanol [MP Biomedicals, 194834], 0.1 U/μL lyticase [Yeasen, 10403ES81], prepared in PBS) at 30°C for 20 min. Spheroplasts were washed genteelly twice in sorbitol buffer. Centrifugation steps after cell wall removal were performed at 300 × *g*. In the interim, cover glass was coated by 20 μL 0.1% poly-d-lysine (Sangon-Biotech, E607014) and allowed to dry at 37°C for 30 min. Spheroplasts were laid on coated cover glass and let to adhere for 15 min, after which excess cells were gently aspirated. Cover glass carrying spheroplasts was incubated in blocking buffer A (0.1% bovine serum albumin [BSA; Sangon-Biotech; A600332], 1.2 M sorbitol, 0.2% Triton X-100, and 0.05% SDS, prepared in PBS) at room temperature for 10 min, then washed three times with blocking buffer B (0.1% BSA, 1.2 M sorbitol, prepared in PBS) and left in blocking buffer B for 30 min during the last wash. Cover glass carrying spheroplasts was incubated with primary antibody (anti-V5 mouse; Abbkine; A02170) (1:100 in blocking buffer B) prepared in blocking buffer B for at least 2 h. Spheroplasts were washed three times with blocking buffer B. Incubation with secondary antibody (Dylight 488; Invitrogen; 35502) (1:100 in blocking buffer B) was performed in darkness for 1 h. Spheroplasts were then washed three times with blocking buffer B. Residual fluid was carefully aspirated and the cover glass with yeast cells was mounted on a slide with a drop of antifade mounting medium (Invitrogen; S36967). The edge of the cover glass was sealed with nail polish.

### Yeast growth assays.

Plate assays were as follows: 1 optical density at 600 nm (OD_600_) of midlog phase yeast cells were collected and washed once with water. Cells were resuspended in water, and then 10 time serial dilutions were made. Four microliters of of sample was spotted on agar plates and incubated at 30°C for most tests or 25°C and 37°C for *sec7-ts* test. Liquid culture assays were as follows: midlog phase yeast cells were collected, washed once with water, and resuspended in an appropriate testing medium. Cells were then diluted to a starting OD_600_ of 0.02 in the testing medium and incubated in a shaker at 30°C. ODs were monitored by withdrawing a small aliquot of the culture.

### BODIPY/lipolysis assay.

Two optical denisities of midlog phase yeast cells were collected, and starved in SD-N medium for 1 h. Starved cells were collected and resuspended in 200 μL sterile water. One microliter of 100 μg/mL BODIPY (493/503) was added and the cells were incubated for 2 m. Cells were washed with water and resuspended in 4 mL fresh SMD medium and then incubated for 4 h.

### Quantification of light microcopy images: DAB and BODIPY staining.

For mitochondrial DAB staining, the stained areas were first segmented by setting an empirical threshold and then manually edited to remove vacuolar regions of interest (ROIs) and extracellular ROIs. For endoplasmic reticulum DAB staining, the stained areas were manually marked. For BODIPY staining, the stained dots were segmented by training the Weka plugin in ImageJ (23). The resulting ROIs were quantified using the “Analyzing Particles” tool in ImageJ (24, 25).
