# Supplementary material for: A Validated Set of Ascorbate Peroxidase-Based Organelle Markers for Electron Microscopy of Saccharomyces cerevisiae
Source: mSphere. 2022 Jun 21;7(4):e00107-22. doi: 10.1128/msphere.00107-22 (PMC9429943; doi:10.1128/msphere.00107-22)
Supplement: TABLE S1 [file msphere.00107-22-s0001.pdf]

Table S1. Plasmid Set I*.

Plasmid	Parental Plasmid	Inserted Fragments	Restriction Sites	Primers	Templates /Sources	Linearization Site
ClhN-4V5-APEX2-URA	ClhN-4V5-URA3(K.l.)	APEX2	AvrII	CCTAGGGGAAAGTCTTACCCAACTGTG, GGCGCGCCTCAAGATCTGCACTGAGCGTCCA	Chemical Synthesis	(N/A)
ClhN-p1k-Pex3-4V5-APEX2-URA	ClhN-4V5-APEX2-URA	Pex3(promoter+ORF)	PacI	GCAGGTCGTGGGATTCCTGGTGTTAATCATTATCCATTTT, CCGCTAGCGATGTTAATTAAAGGCTTGAAGGAAAACGAGC	Genomic DNA	BstBI
ClhN-p1k-Snf7-4V5-APEX2-URA	ClhN-4V5-APEX2-URA	Snf7(promoter+ORF)	PacI	GCAGGTCGTGGGATTCCTGGATGCGTTGATTATTGGGTTT, CCGCTAGCGATGTTAATTAAAAGCCCCATTTCTGCTTGTA	Genomic DNA	BstBI
ClhN-p1k-Tgl3-4V5-APEX2-URA	ClhN-4V5-APEX2-URA	Tgl3(promoter+ORF)	PacI	GCAGGTCGTGGGATTCCTGGGACTGGCTGATAGCCTCGAC, CCGCTAGCGATGTTAATTAACCTACTCCGTCTTGCTCTTA	Genomic DNA	BstBI
ClhN-p1k-Sec7-4V5-APEX2-URA	ClhN-4V5-APEX2-URA	Sec7(promoter+ORF)	PacI	GCAGGTCGTGGGATTCCTGGAATGTTGAGATAATTGTTGG, CCGCTAGCGATGTTAATTAAATCAGTAGAAAGGTATAATT	Genomic DNA	KasI

ClhN-p1k-Cox4-V5-APEX2-Ura	ClhN-URA3(K.l.)	Cox4(promoter+ORF), APEX2	AvrII	GGCCGCCAGCTGAAGCTTCGGATTGGCCCAATTGCTGC, CAAACCCAACAATGGATTTGGAATTGGTTTACCGTGATGGTGGTCATCATTTGG, CCAAATCCATTGTTGGGTTTGGATTCTACTGGAAAGTCTTACCCAACTGTG, CTCACCTAGGCTAAGATCTGCACTGAGCGTCCAGGG	Genomic DNA, ClhN-4V5-APEX2-URA	BstBI
ClhN-p1k-Cox4-GFP-APEX2-Ura	ClhN-4V5-APEX2-URA	Cox4-GFP	BsiWI	GGCCGCCAGCTGAAGCTTCGGATTGGCCCAATTGCTGC, GGAATCCCACGACCTGCAGCTTTGTACAATTCATCCATACC	Cox4-GFP-Ura(Zhu et al., 2019)	BstBI
ClhN-pADH3-4V5-APEX2-URA	ClhN-4V5-APEX2-URA	Adh3 promoter	BsiWI, NheI	CGGCCGCCAGCTGAAGCTTCGTACGTCCTTACGAAAAAAAAGATC, GATTTGGAATTGGTTTACCGCTAGCGATGGTGTTAACTTATGACT	Genomic DNA	(N/A)
ClhN-pADH3-Pex3-4V5-APEX2-URA	ClhN-pADH3-4V5-APEX2-URA	Pex3 ORF	NheI	AGTCATAAGTTAACACCATCATGGCCCCAAATCAAAGATC, GGAATTGGTTTACCGCTAGCAGGCTTGAAGGAAAACGAGC	Genomic DNA	SphI

ClhN-pADH3-Snf7-4V5-APEX2-URA	ClhN-pADH3-4V5-APEX2-URA	Snf7 ORF	NheI	AGTCATAAGTTAACACCATCATGTGGTCATCACTTTTTGG, GATTTGGAATTGGTTTACCGCTAGCAAGCCCCATTTCTGCTTGTA	Genomic DNA	SphI
ClhN-pADH3-Tgl3-4V5-APEX2-URA	ClhN-pADH3-4V5-APEX2-URA	Tgl3 ORF	NheI	AGTCATAAGTTAACACCATCATGAAGGAAACGGCGCAGGA, GATTTGGAATTGGTTTACCGCTAGCCCTACTCCGTCTTGCTCTTA	Genomic DNA	SphI
ClhN-pADH3-Sec7-4V5-APEX2-URA	ClhN-pADH3-4V5-APEX2-URA	Sec7 ORF	NheI	AGTCATAAGTTAACACCATCATGTCTGAACAGAATTCAGT, GATTTGGAATTGGTTTACCGCTAGCATCAGTAGAAAGGTATAATT	Genomic DNA	SphI
ClhN-pERG6-Pex3-4V5-APEX2-URA	ClhN-p1k-Erg6-V5-APEX2-URA	Pex3 ORF	NheI	AATATAGTAGGCAGCATAAGCGTACGATGGCCCCAAATCAAAGATC, GATTTGGAATTGGTTTACCGCTAGCAGGCTTGAAGGAAAACGAGC	Genomic DNA	SphI
ClhN-pLSP1-Pex3-4V5-APEX2-URA	ClhN-pLSP1-4V5-APEX2-URA	Pex3 ORF	NheI	TTCAAAGCTCCAACACAAGACGTACGATGGCCCCAAATCAAAGATC, GATTTGGAATTGGTTTACCGCTAGCAGGCTTGAAGGAAAACGAGC	Genomic DNA	SwaI

* Plasmid backbones were obtained by restriction enzyme digestion of parental plasmids.
