# Supplementary material for: A Validated Set of Ascorbate Peroxidase-Based Organelle Markers for Electron Microscopy of Saccharomyces cerevisiae
Source: mSphere. 2022 Jun 21;7(4):e00107-22. doi: 10.1128/msphere.00107-22 (PMC9429943; doi:10.1128/msphere.00107-22)
Supplement: TABLE S2 [file msphere.00107-22-s0002.pdf]

Table S2. Plasmid Set II#.

Plasmid	Primers for Plasmid Backbone	Templates/ Sources	Primers for Inserted Fragment	Templates /Sources	Linearization Site
ClhN-p1k-Erg6-V5-APEX2-URA	GGTAAACCAATTCCAAATCC, GAAGCTTCAGCTGGCGGCCG	ClhN-p1k-Cox4-V5-APEX2-Ura	CGGCCGCCAGCTGAAGCTTCTTACTATTTTTGAAAAGATG, GGATTTGGAATTGGTTTACCTTGAGTTGCTTCTTGGGAAG	Genomic DNA	SphI
ClhN-p1k-Vrg4-V5-APEX2-URA	GGTAAACCAATTCCAAATCC, GAAGCTTCAGCTGGCGGCCG	ClhN-p1k-Cox4-V5-APEX2-Ura	CGGCCGCCAGCTGAAGCTTCACTCAATAGACATTCTAAAG, GGATTTGGAATTGGTTTACCTTTACGTAAAGGTTGGGCTT	Genomic DNA	BmgBI
ClhN-p1k-Elo3-V5-APEX2-URA	GGTAAACCAATTCCAAATCC, GAAGCTTCAGCTGGCGGCCG	ClhN-p1k-Cox4-V5-APEX2-Ura	CGGCCGCCAGCTGAAGCTTCGATACAGTTGCGTCGTTAGA, GGATTTGGAATTGGTTTACCAGCTTTCCTGGAAGAGACCT	Genomic DNA	BstBI
ClhN-p1k-OM14-V5-APEX2-Ura	GGTAAACCAATTCCAAATCC, GAAGCTTCAGCTGGCGGCCG	ClhN-p1k-Cox4-V5-APEX2-Ura	CGGCCGCCAGCTGAAGCTTCCACGCAACCAAATATGTAAG, GGATTTGGAATTGGTTTACCTTTCTTGTCGTATCTGGAGT	Genomic DNA	SmaI
ClhN-pTPI1-Emc1-GFP-APEX2-Ura	TAAACTAAAAATGAAGATAACGTGTACAGAC,	ClhN-p1k-Emc1-GFP-APEX2-Ura	CTGAAGCTTCCCTACGTTAGTGTGAGCGGG, CGTTATCTTCATTTTTAGTTTATGTATGTGTTTTTTG	Genomic DNA	SphI

	CTAACGTAGGGAAGCTTCAGCTGG CGGCCG				
ClhN-pTPI1-Cox4-GFP-APEX2-Ura	CATAAACTAAAAATGCTTTCACTA CGTCAATC, CTAACGTAGGGAAGCTTCAGCTGG CGGCCG	ClhN-p1k-Cox4-GFP-APEX2-Ura	CTGAAGCTTCCCTACGTTAGTGTGAGCGGG, GTGAAAGCATTTTTAGTTTATGTATGTGTT	Genomic DNA	SphI
ClhN-pTPI1-Emc1-flag-APEX2-Ura	GATTATAAAGATGATGATGATAAA GCTGCAGGTCGTGGGATTCCTGGG, CATCATCATCTTTATAATCTTTAAT TAGCCATTGGGATTTCAACT	ClhN-pTPI1-Emc1-GFP-APEX2-Ura	(N/A)	(N/A)	SphI
ClhN-pTPI1-Cox4-flag-APEX2-Ura	GATTATAAAGATGATGATGATAAA GCTGCAGGTCGTGGGATTCCTGGG, TCATCATCATCTTTATAATCGTGAT GGTGGTCATCATTTGGAACAC	ClhN-pTPI1-Cox4-GFP-APEX2-Ura	(N/A)	(N/A)	SphI

Plasmid backbones were obtained by PCR amplification of parental plasmids.
