# Supplementary material for: A Validated Set of Ascorbate Peroxidase-Based Organelle Markers for Electron Microscopy of Saccharomyces cerevisiae
Source: mSphere. 2022 Jun 21;7(4):e00107-22. doi: 10.1128/msphere.00107-22 (PMC9429943; doi:10.1128/msphere.00107-22)
Supplement: TABLE S3 [file msphere.00107-22-s0003.pdf]

Table S3. Plasmid Set III [%]

Plasmid	Source Plasmids	Fragments	Restriction Sites	Linearization Site(s)
P_{COX4}-Cox4-DuDre-APEX2·URA3(K.l.)	ClhN-4V5-APEX2-URA	Plasmid backbone	AatII, AvrII	BstBI
	OM64	P_{COX4}-Cox4-DuDre	AatII, AvrII	
OM14- 4V5-APEX2-KT209-ter	OM14-2EGFP-KT209-ter	Plasmid backbone	AscI, PacI	NgoMIV and SphI
	ClhN-4V5-APEX2-URA	4V5-APEX2	AscI, PacI	

[%] Constructed by enzymatic digestion of source plasmids and subsequent ligation of resulting fragments.
