# Supplementary material for: A Validated Set of Ascorbate Peroxidase-Based Organelle Markers for Electron Microscopy of Saccharomyces cerevisiae
Source: mSphere. 2022 Jun 21;7(4):e00107-22. doi: 10.1128/msphere.00107-22 (PMC9429943; doi:10.1128/msphere.00107-22)
Supplement: TABLE S4 [file msphere.00107-22-s0004.pdf]

Table S4. Strains used in this study.

Name	Genotype
TN124	*MATα leu2-3,112 trp1 ura3-52 pho8::pho8Δ60 pho13::LEU2*
YCH722	TN124 *PCOX4:: PCOX4-COX4-GFP-APEX2·URA3(K.l.)*
YCH760	TN124 *PTPI1:: PTPI1-EMC1-flag-APEX2·URA3(K.l.)*
YCH761	TN124 *PTPI1:: PTPI1-COX4-GFP-APEX2·URA3(K.l.)*
YCH762	TN124 *PTPI1:: PTPI1-EMC1-GFP-APEX2·URA3(K.l.)*
YCH763	TN124 *PTPI1:: PTPI1-COX4-flag-APEX2·URA3(K.l.)*
YCH770	TN124 *PCOX4:: PCOX4-COX4-V5-APEX2·URA3(K.l.)*
YCH771	TN124 *POM14:: POM14-OM14-V5-APEX2·URA3(K.l.)*
YCH790	YRD12 *PERG6:: PERG6-ERG6-mCherry·TRP(K.l.)*
YCH791	YRD12 *PPEX3:: PPEX3-PEX3-DuDre·TRP(K.l.)*
YCH792	YRD12 *PVPS4:: PVPS4-VPS4-DuDre·TRP(K.l.)*
YCH793	YRD12 *PSEC7:: PSEC7-SEC7-DuDre·TRP(K.l.)*
YCH794	YRD12 *PANP1:: PANP1-ANP1-mCherry·TRP(K.l.)*
YRD01	TN124 *PERG6:: PERG6-ERG6-V5-APEX2·URA3(K.l.)*
YRD02	TN124 *PVRG4:: PVRG4-VRG4-V5-APEX2·URA3(K.l.)*
YRD03	TN124 *PELO3:: PELO3-ELO3-V5-APEX2·URA3(K.l.)*
YRD06	TN124 *PADH3:: PADH3-SNF7-4V5-APEX2·URA3(K.l.)*
YRD07	TN124 *PADH3:: PADH3-TGL3-4V5-APEX2·URA3(K.l.)*
YRD08	TN124 *PADH3:: PADH3-SEC7-4V5-APEX2·URA3(K.l.)*
YRD11	TN124 *PERG6:: PERG6-PEX3-4V5-APEX2·URA3(K.l.)*
YRD12	TN124 *PLSP1:: PLSP1-PEX3-4V5-APEX2·URA3(K.l.)*
YRD14	YRD01 *PTGL3:: PTGL3-TGL3-mCherry·TRP(K.l.)*
YRD15	YRD01 *PANP1:: PANP1-ANP1-mCherry·TRP(K.l.)*
YRD16	YRD01 *PSEC7:: PSEC7-SEC7-DuDre·TRP(K.l.)*
YRD17	YRD01 *PVPS4:: PVPS4-VPS4-DuDre·TRP(K.l.)*
YRD18	YRD01 *PPEX3:: PPEX3-PEX3-DuDre·TRP(K.l.)*
YRD20	YRD02 *PANP1:: PANP1-ANP1-mCherry·TRP(K.l.)*
YRD21	YRD02 *PERG6:: PERG6-ERG6-mCherry·TRP(K.l.)*
YRD22	YRD02 *PSEC7:: PSEC7-SEC7-DuDre·TRP(K.l.)*
YRD23	YRD02 *PVPS4:: PVPS4-VPS4-DuDre·TRP(K.l.)*
YRD24	YRD02 *PPEX3:: PPEX3-PEX3-DuDre·TRP(K.l.)*
YRD35	YRD06 *PSNF7:: PSNF7-SNF7-mCherry·TRP(K.l.)*
YRD36	YRD06 *PERG6:: PERG6-ERG6-mCherry·TRP(K.l.)*
YRD37	YRD06 *PANP1:: PANP1-ANP1-mCherry·TRP(K.l.)*
YRD38	YRD06 *PSEC7:: PSEC7-SEC7-DuDre·TRP(K.l.)*
YRD39	YRD06 *PPEX3:: PPEX3-PEX3-DuDre·TRP(K.l.)*
YRD45	YRD08 *PSEC7:: PSEC7-SEC7-DuDre·TRP(K.l.)*
YRD46	YRD08 *PERG6:: PERG6-ERG6-mCherry·TRP(K.l.)*
YRD47	YRD08 *PANP1:: PANP1-ANP1-mCherry·TRP(K.l.)*
YRD48	YRD08 *PVPS4:: PVPS4-VPS4-DuDre·TRP(K.l.)*
YRD49	YRD08 *PPEX3:: PPEX3-PEX3-DuDre·TRP(K.l.)*

YRD50	TN124 *PSNF7:: PSNF7-SNF7-4V5-APEX2·URA3(K.l.)*
YRD51	TN124 *PTGL3:: PTGL3-TGL3-4V5-APEX2·URA3(K.l.)*
YRD52	TN124 *PSEC7:: PSEC7-SEC7-4V5-APEX2·URA3(K.l.)*
YRD53	TN124 *PPEX3:: PPEX3-PEX3-4V5-APEX2·URA3(K.l.)*
YRD54	TN124 *PADH3:: PADH3-PEX3-4V5-APEX2·URA3(K.l.)*
BY4741	*MATa his3Δ1 leu2Δ0 LYS2 met15Δ0 ura3Δ0*
YZT908	BY4741 *elo3Δ*
YMJ405	BY4741 *snf7Δ::HIS5(S.p.)*
LYAP1	YZT908 *PELO3:: PELO3-ELO3-V5-APEX2·URA3(K.l.)*
LYAP2	*sec7-1 PADH3:: PADH3-SEC7-4V5-APEX2·URA3(K.l.)*
LYAP3	BY4741 *PLSP1:: PLSP1-PEX3-4V5-APEX2·URA3(K.l.)*
LYAP4	BY4741 *PVRG4:: PVRG4-VRG4-V5-APEX2·URA3(K.l.)*
LYAP5	BY4741 *OM14-4V5-APEX2·URA3*
LYAP6	YMJ405 *PADH3:: PADH3-SNF7-4V5-APEX2·URA3(K.l.)*
LYAP7	*tgl3Δ PADH3:: PADH3-TGL3-4V5-APEX2·URA3(K.l.)*
LYAP8	TN124 *PCOX4:: PCOX4-COX4-DuDre-APEX2·URA3(K.l.)*
